# A Hierarchical Communication Architecture for Oceanic Surveillance Applications

**DOI:** 10.3390/s111211343

**Published:** 2011-11-30

**Authors:** Elsa Macias, Alvaro Suarez, Francesco Chiti, Andrea Sacco, Romano Fantacci

**Affiliations:** 1 Grupo de Arquitectura y Concurrencia (GAC), Departamento de Ingeniería Telemática, Universidad de Las Palmas de Gran Canaria, Campus Universitario de Tafira, 35017 Las Palmas de Gran Canaria, Gran Canaria, Spain; E-Mail: asuarez@dit.ulpgc.es (A.S.); 2 Dipartimento Elettronica e Telecomunicazioni, University of Florence, Florence 50139, Italy; E-Mails: francesco.chiti@unifi.it (F.C.); andrea.sacco85@gmail.com (A.S.); romano.fantacci@unifi.it (R.F.)

**Keywords:** multimedia streaming, underwater sensor networks, MAC, WiFi, Zigbee, acoustic distributed surveillance

## Abstract

The interest in monitoring applications using underwater sensor networks has been growing in recent years. The severe communication restrictions imposed by underwater channels make that efficient monitoring be a challenging task. Though a lot of research has been conducted on underwater sensor networks, there are only few concrete applications to a real-world case study. In this work, hence, we propose a general three tier architecture leveraging low cost wireless technologies for acoustic communications between underwater sensors and standard technologies, Zigbee and Wireless Fidelity (WiFi), for water surface communications. We have selected a suitable Medium Access Control (MAC) layer, after making a comparison with some common MAC protocols. Thus the performance of the overall system in terms of Signals Discarding Rate (SDR), signalling delay at the surface gateway as well as the percentage of true detection have been evaluated by simulation, pointing out good results which give evidence in applicability’s favour.

## 1. Introduction

*Wireless Sensor Networking* (*WSN*) is a recently introduced paradigm making possible the monitoring of complex systems using low cost easily deployable devices. The monitored phenomena constraints the design of the sensors, while the features of the environment in which they operate imposes restrictions to the communication devices design. Finally, both these aspects drive the communication strategy between WSN and the Central Server (CS). WSN has been applied to many applications. Among them, one of the most important applications is sensor data collection (e.g., water monitoring). In [1] the authors present a survey on recent advances in sensor data collection research area.

A novel application field is represented by *Under Water* WSN (*UWSN*) [2] which usually faces harsh communications conditions like large propagation delays, relatively large motion-induced Doppler effects, high bit-error probability and very limited bandwidth [3]. At present, the UWSNs include complex and expensive devices to support the sensing: they generally include only acoustic communication modems [4] (defining the so called *Under Water Acoustic Sensor Network* (*UW-ASN*)) and only provide the sensing of certain physical parameters.

When designing *Multimedia* UWSN applications [5] two different strategies can be adopted: (a) distributed video cameras connected to sensors that monitor physical properties of the environment, (b) cameras acting as sensors sending directly multimedia information to CS. In both cases CS processes multimedia information and alarms. The monitoring process can be time *continuous* or *discontinuous*. Usually a discontinuous process is required for underwater monitoring process due to some difficulties with off line data fusion [6]. This avoids the usage of *streaming techniques* to issue video information directly from the underwater cameras to the CS. The underwater cameras must be included in a mobile device which must emerge to the water surface in order to wirelessly send the video information to the CS. The authors in [7] present techniques for a typical sensor node can be upgraded to a camera sensor node by attaching a standard flash memory (standard SD card), a small low-cost camera, and a software update. The C-code software for the techniques introduced in [7] is freely available.

Some of the key features needed to design an efficient UWSN, and still not jointly addressed in the literature, are:

- Use of low power underwater sensors [8].
- Optimization of the communication interfaces according to the medium characteristics.
- Optimization of the network and especially the MAC layer [9].

**Figure 1.**
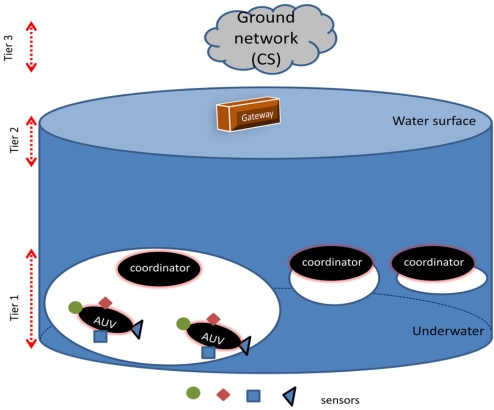
Schema of the three tier system architecture.

The objective of this paper is to present the design of a multimedia distributed monitoring system for UWSNs, exploiting available technologies and to evaluate system performance with a realistic underwater channel model by simulation. To the best of our knowledge, this problem has not been already addressed to this integrated application-driven approach. To this end, it is investigated a UWSN three tier architecture comprised of a CS in the Ground network, a gateway in the surface of the water and sensor nodes (Figure 1)—deployed in an *Autonomous Underwater Vehicle* (AUV)—each of them is equipped with:

- Standard Charge-Coupled Device (CCD) camera for recording special underwater events.
- Standard signal processor and memory to store multimedia information.
- Low cost acoustic modems, as defined in [4].
- A Zigbee or a WiFi Wireless Network Card Interface to transmit to a gateway in the surface of the water, taking advantages of previous results concerning video streaming information [10]. The gateway issues information to CS using a cellular link. Using multimedia sensor nodes in WSN is possible as it is shown in [11] and some issues and challenges should be considered as the ones outlined in [10]. In [12] is presented a survey of wireless video sensor node platforms (WVSNP). The authors propose the design of a novel WVSNP. One important issue in the design is the usage of a dual radio system (WiFi-Zigbee) for flexible video streaming. The design is still in its early stages.

As the number of alarms overcomes a proper threshold, AUVs *emerge* and *upload* multimedia information to CS using a WiFi or Zigbee connection. The critical part of our system is this management of alarms. A suitable MAC might be adopted in order to achieve the time constraints associated to those alarms. To this end we have compared the more promising candidates with a good trade-off between complexity and performance, pointing out Unsynchronized T-Lohi protocol. In addition, the proper *flavour* [13] has been tuned achieving the best performance in terms of collision rate, throughput and latency [14]. We are not concerned with the problems (waves, strong water reflexions...) that arise in the communication in the surface of the water using wireless channels. We experimentally have tested that in the case of regular flat water surface (swimming pool), Zigbee or WiFi exhibit a sufficient performance [15]. We suppose the bandwidth provided by the telecommunication operator for the cellular link is enough to our purposes.

The structure of the remaining part of this paper is the following: Section 2 deals with an overview of the state of the art for UW-ASN, the system architecture is presented in Section 3, numerical results derived with computer simulations are provided in Section 4 and, finally, conclusions are drawn in Section 5.

## 2. Overview of UW-ASN

In recent research papers [16] UWSNs have been divided into two main categories: the UW-ASN and the *Under Water Hybrid Sensor Network* (UW-HSN). Both differ in some specific aspects. In particular, UW-ASNs are all-acoustic, small sensors networks. They are composed of a limited number of sensors monitoring a large area and use the acoustic waves as a communication mean. The usage of these waves presents some advantages and disadvantages. The main advantage is that with a low number of sensors, it is possible to monitor a wide area since the coverage radius is in the order of kilometres. The underwater sound speed is approximately 1,500 mps, which is about 200,000 times lower than the speed of the light in air. This leads to large propagation delays and relatively large motion-induced Doppler effects. Phase and amplitude fluctuations lead to a high bit-error probability relative to most radio channels, requiring *Forward Error Correction* (*FEC*) coding. In addition, the acoustic channel has strong attenuation as the frequency increases [17], leading to very limited bandwidth. Due to these problems, *ad-hoc* protocols are needed for this kind of networks as discussed in [18]. Moreover the hardware devices used for acoustic communications, that are acoustics modems, are typically bulky, costly and power consuming.

Recently the *Woods Hole Oceanographic Institution* (WHOI) has developed a research oriented underwater acoustic modem [4] that resolves much of the mentioned problems. Other examples of low-power, low-cost, *ad-hoc* acoustic modems are presented in [19] and [20].

UW-HSN instead, are *hybrid* networks that use EM/optical waves for short range high data rates communications and acoustic waves for long range low data rates communications [20]. In these networks the sensors are densely deployed in the area of interest and can adopt different approaches for the communication method. One of the most efficient methods for long-term monitoring networks is the *data muling* as described in [21]. This approach integrates tiny sensors (with no navigation capabilities) and AUVs. The AUVs navigate over the cluster [6] collecting data and then transmitting them back to a local base station. The sensors can use either high bandwidth optical links (in green-blue wavelength) or EM wireless links. This is possible because the AUVs could travel in the proximity of the sensors, allowing for a very short range high bandwidth communications. This anyway requires that AUV is able to localize the sensors, using a proper ranging technique as for instance resorting to optical waves as described in [22]. Several others methods are being investigated for UW-ASN localization in [23–25], as such as more realistic models (taking into account ocean currents) of the deployment environment in [26].

## 3. System Architecture

**Figure 2.**
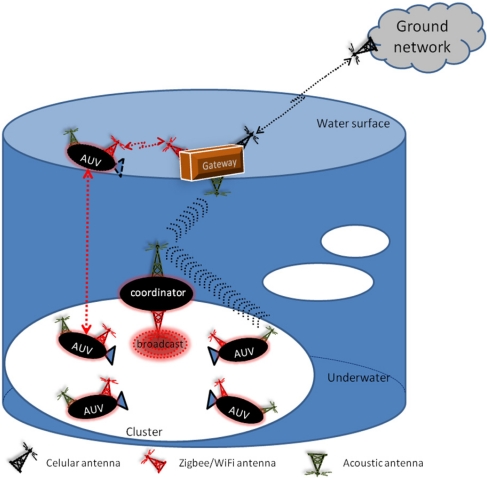
Network topology and organization.

The network topology and components organization of the system is presented in Figure 2. There are at least four AUVs equipped with different types of multimedia sensors: temperature, visible-field camera, infrared-field camera, microphone, and so on. AUVs monitor a specified area of interest performing data fusion, features detection, temporary storing data in an internal memory. Each sensor has associated an alarm event that it is raised in case a predefined condition is arisen. The AUVs are provided with two different antennas technologies: Zigbee (WiFi) and acoustic. The gateway is provided with a three different antennas: Zigbee to communicate with an AUV when it is in the water surface, an acoustic antenna and a microwave antenna to communicate with the network in the Ground (ground network).

The surroundings of the zone to be monitored are divided in several regions (marked as white ellipses in Figure 2). We define a cluster of AUVs as those AUVs monitoring the same underwater region, with a cluster head (coordinator). The coordinator communicates with the AUV and the gateway using the acoustic antenna while it is under the water. Eventually, when it is in the surface of the water it will communicate with the gateway using its Zigbee antenna.

**Figure 3.**
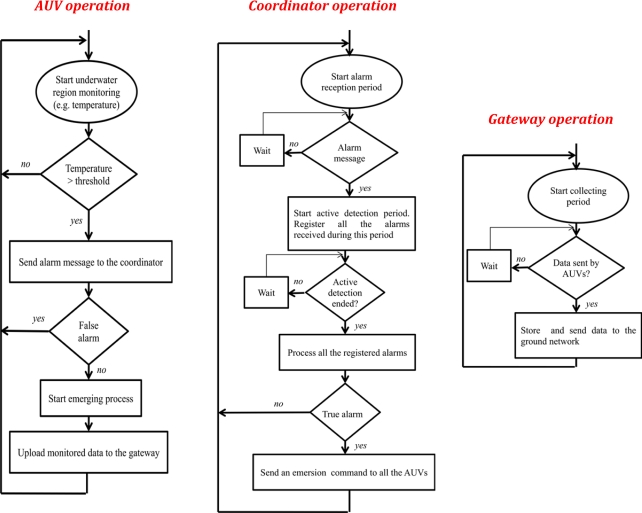
System operation specified for each main component.

The operation of each component is presented in Figure 3.

- An AUV is in charge of monitoring part of a region controlled by the coordinator. For example, if different AUVs are observing that region with a camera, the different cameras will be aligned in order to cover the global region (with overlapped zones). A cycle of the operation of an AUV consists in the following main actions:
  ○ It starts monitoring a part of the underwater region.
  ○ If a predefined condition arises on a particular sensor then it will send an alarm message to the coordinator; otherwise it will start again a new monitoring period.
  ○ If it receives (from the coordinator using the acoustic antenna) an emerging command it will emerge to the water surface. It will upload the monitored data (using the Zigbee antenna) into the gateway and it will start again a new monitoring period. In Figure 3 we abstract this in a decision box that verifies if a false alarm will be produced and a box indicating the AUV must emerge to the water surface. AUVs localization (an interesting survey is presented in [27]) can be used for data tagging, node tracking and target detection. In our system the AUVs only move vertically to emerge or submerge. For this reason they do not need to know their position since we do not consider other kind of underwater movements.
- The coordinator starts waiting for at least an alarm message from the AUVs. Once an alarm message is received it will start a period of active detection to register all the alarms, while processing them at the end of the monitoring period. The cluster coordinator discriminates between a true or false alarm according to Equation (1), where *n_a_* is the number of received alarms and *N* is the number of coordinated AUVs:
  (1) $$\begin{array}{l} n_{a}\geq \frac{N}{2}\to \mathrm{TRUE}\;\mathrm{ALARM}\\ n_{a}<\frac{N}{2}\to \mathrm{FALSE}\;\mathrm{ALARM} \end{array}$$
  ○ If the coordinator detects a false alarm then it will start a new period of alarm detection; otherwise, it will send an emersion command to all the AUVs belonging to its cluster (The emerging process of the AUVs will be decided by the coordinator in order not to leave the zone free of survey; that is, at least three AUVs must be surveying the zone. Remember that there are at least four AUVs within the cluster).
- The gateway starts a collecting period waiting for the AUVs to send it the registered data (for example, a video of the part of the region they monitored in which an oil leak was produced). It immediately sends that data to the CS in the ground network using the microwave antenna, for further analysis.

**Figure 4.**
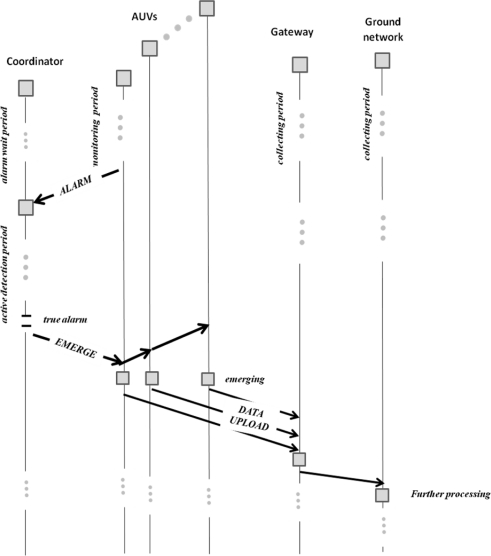
Protocol actions among components.

In Figure 4 we show a schema of the basics actions of the communication protocol among the coordinator, the AUVs, the gateway and the ground network.

The initialization of the protocol consists of: (a) the coordinator is waiting for an alarm message from the AUVs; (b) The AUVs start their monitoring process; (c) The gateway is slept (it will be awaked by the messages of the AUVs once they will be in the water surface). The server that will do further processing is always slept. After a waiting period, the coordinator will receive alarm messages from the AUVs, it will test a true alarm and then it will send an emerge message to the AUVs. The AUVs will emerge and will awake the gateway uploading the monitored data. Then the gateway will send the data to the Server in the ground network.

### Technological Choices

Regarding the acoustic transducer, we have used the WHOI Micro Modem with Frequency Hopped-Frequency Shift Keyed (*FH-FSK*) modulation. The packet size is 32 bits, as well as the frame size. The data-rate of the Micro Modem with this kind of modulation is 80 bps, thus the duration of the packet (frame) is about 3.2 s. The central frequency of communication is set to 10 kHz with a bandwidth of 4 kHz. This corresponds to the band A of the Micro Modem.

**Figure 5.**
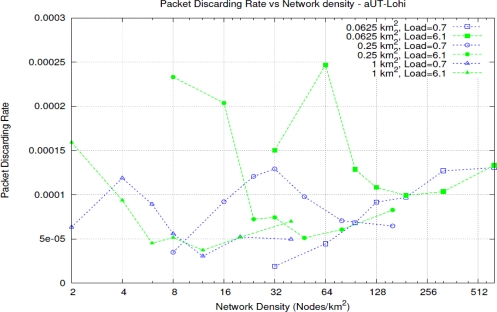
Packet Discarding Rate *versus* Node Density, for different network sizes and different offered loads.

Regarding the MAC protocol, we have made a preliminary comparison between three alternatives. A simple Aloha, an Aloha with Contention Window [28] and an Unsynchronized T-Lohi protocol in Aggressive flavour [13]. The latter one has shown the best performance in terms of collision rate, throughput and latency. For the Unsynchronized T-Lohi protocol in Aggressive flavour Figure 5 shows the *Packet Discarding Rate* (*PDR*) for different node density values due to errors and collisions.

**Figure 6.**
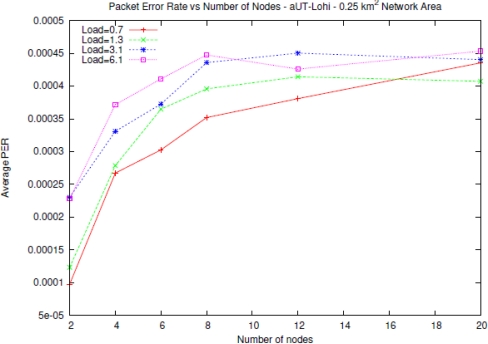
Average Packet Error Rate *versus* network size, for different offered loads.

Let us note the PDR decreases with an increasing number of nodes till a minimum value. All the preliminary tests have been made with ideal conditions (ideal transducer and ideal medium). Testing the T-Lohi protocol with realistic conditions, like non-ideal medium and transducer, has lead to a drastic decrease in performance. As shown in Figure 6, the average *Packet Error Rate* (*PER*) for the T-Lohi protocol does not decrease, as in the ideal case, with the number of nodes but instead it seems to reach a “saturation” value. The medium simulation has been made using data coming from the BELLHOP algorithm [26].

## 4. Simulation Results

We used UWSN module of NS-3 [29] with the simulation parameters shown in Table 1.

All the graphs shown are function of average detection interval. The detection interval is the time elapsing between two consecutive detection events (The detection events are fired at random time following a uniform distributed random distribution). The *Signal Discarding Rate* (*SDR*) is defined as the ratio of erroneous or lost signals (warnings messages) compared to the total sent signals. The *Surface Gateway Signalling Delay* (*SGSD*) is defined as the time elapsed from the detection by the AUV of an event of interest and the completion of the data upload to the Surface Gateway. The components of the SGSD are shown in Equation (2):

(2) $$\Delta =t_{\mathrm{tx}}+t_{\mathrm{proc}}+t_{\mathrm{msg}}+t_{\mathrm{surf}}+t_{\mathrm{up}}$$

where:

1. *t_tx_* is the warning message transmission time,
2. *t_proc_* is the coordinator active detection duration,
3. *t_msg_* is the emerging message transmission time,
4. *t_surf_* is the AUV surfacing time, and
5. *t_up_* is the data upload time.

**Figure 7.**
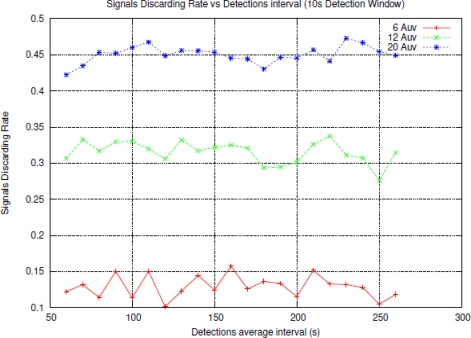
Signal Discarding Rate against detection interval, for different number of nodes.

**Figure 8.**
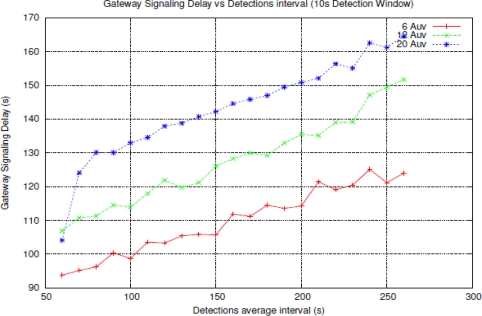
Average Surface Gateway signalling delay against detection interval, for different number of nodes.

As shown in Figure 7 the rate of lost signals increments with the number of nodes into the network since the larger number of nodes the greater collision rate and discarding packets but the rate of lost signals is independent from the detection interval. Regarding the SGSD (Figure 8), there is also an increment with the number of nodes. These results can be expected from Equation (2) where all the components increase with the number of nodes excepting the AUV surfacing time that is equal to the AUV speed (We have estimated an emerging speed of 2.3 mps, basing on common AUV speed). The different number of nodes only incurs in a widening of the *active detection* period and then in an “offset” time added to the emerging time.

As the number of nodes increases, the absolute number of signals needed for a *true detection* increases too according to Equation (1). As we shown in Figure 7 the larger number of nodes the greater SDR, then *True Detection Rate* decreases. To compensate the effect of a larger SDR, it could be widen the *active detection* period, with the side effect of lowering the reactivity of the whole system. In fact, initially the *active detection* period was fixed to 20 s but, looking at the results, we decided to make it “adaptive” to the number of coordinated nodes, that is to say, the number of AUVs.

**Figure 9.**
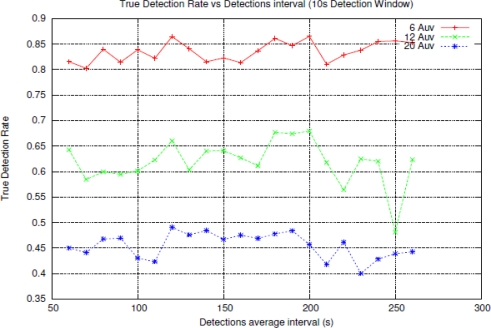
Percentage of True Detection against detection interval, for different number of nodes.

The results shown in Figure 9 are for an *active detection* duration of 3*N_coord_* seconds, where *N_coord_* is the number of coordinated nodes. In a real case, during the design process of the application, the simulation results can be exploited to fine-tune the signalling delay against the percentage of true detections, and hence make the whole system more reactive or reliable.

## 5. Conclusions

The contribution of the paper is mainly focused on a system architecture design supporting distributed sensing to detect anomalous situations occurring in underwater environments. Due to the specific propagation conditions a three tier architecture has been proposed and applied to a real life inspired case study. Nodes functionalities and interfaces have been characterized. In addition, to figure out the overall performance, three different MAC protocols were evaluated out of the scope of this paper and the T-Lohi MAC protocol was selected and tuned taking into account a specific UW-ASN application. Finally, the overall system performance has been validated by means of numerical simulations which take into account real world parameters and models, as well as features of current standard devices.

We have emphasized how the introduction of realistic conditions for the communication channel and hardware devices add losses that have not being considered during the theoretical study. For example, the T-Lohi protocol supplied very good performance but its implementation in real conditions degraded dramatically the performance. However, the results obtained from the simulations were good enough for real-world applications considering *true detection* percentages and reaction times.

Further research must be done in the simulation of the better MAC including dynamic clustering schemes in which the cluster head is elected basing alternatively on network life-time and latency optimization on relying on a semantic approach in order to track a time varying phenomenon.

We are simulating strong conditions in the water surface in order to optimize the disruptions of the WiFi/Zigbee link in the water surface. We are introducing new 3D movement models in NS-3 and realistic models that explain the movement of water. Strong wave in the water surface provokes hard disruptions. The aim of this new work is to complete our mathematical model to optimize the streaming of information in the water surface from the coordinators to the gateway.

As we have stated, streaming on the water surface is feasible using a WiFi or Zigbee antenna. It is very important to estimate the startup time of the streaming (the time that elapses from the detection by the AUV of an event of interest, the emerging time and the synchronization time between the AUV and the gateway to start the upload). The larger startup time, the larger answer to an alarm since the data upload can be only initiated after the startup time. Future work includes a study of streaming startup time hiding.

Finally, a more accurate method to distinguish between true or false alarms must be researched to improve our method that relies on a simple threshold. The method should also take into account that sometimes only some AUVs can be in the right position to detect some alarms whereas the remainder AUVs can be far away from to the target to be monitored.

## Figures and Tables

**Table 1.** Simulation parameters.

**Parameter**	**Value**
Simulation time	36,000 s
Network area	1,000 m^2^
AUVs depth	140 m
Coordinator depth	70 m
Number of times each simulation result is run (we show the average of each simulation)	20
